# Leptin and the rs2167270 Polymorphism Are Associated with Glycemic Control in Type Two Diabetes Mellitus Patients on Metformin Therapy

**DOI:** 10.3390/medicina59050997

**Published:** 2023-05-22

**Authors:** Mahmoud A. Alfaqih, Mukhallad Aljanabi, Ebaa Ababneh, Mariam Khanfar, Mohammad Alqudah, Mai Sater

**Affiliations:** 1 Department of Biochemistry, College of Medicine and Medical Sciences, Arabian Gulf University, Manama 15503, Bahrain; maiss@agu.edu.bh; 2 Department of Physiology and Biochemistry, Faculty of Medicine, Jordan University of Science and Technology, Irbid 22110, Jordan; mukmoh@just.edu.jo (M.A.); eyababneh@just.edu.jo (E.A.); mariamkhanfar94@gmail.com (M.K.); 3 Department of Physiology, Faculty of Medicine, College of Medicine and Medical Sciences, Manama 15503, Bahrain; mohammada@agu.edu.bh

**Keywords:** diabetes mellitus, glycemic control, leptin, single nucleotide polymorphism

## Abstract

*Background and Objectives*: Type two diabetes mellitus (T2DM) is a chronic disease with debilitating complications and high mortality. Evidence indicates that good glycemic control delays disease progression and is hence a target of disease management protocols. Nonetheless, some patients cannot maintain glycemic control. This study aimed to investigate the association between serum leptin levels and several SNPs of the *LEP* gene with the lack of glycemic control in T2DM patients on metformin therapy. *Materials and Methods*: In a hospital-based case-control study, 170 patients with poor glycemic control and 170 patients with good glycemic control were recruited. Serum leptin was measured. Patients were genotyped for three SNPs in the *LEP* gene (rs7799039, rs2167270, and rs791620). *Results*: Serum leptin was significantly lower in T2DM patients with poor glycemic control (*p* < 0.05). In multivariate analysis, serum leptin levels significantly lowered the risk of having poor glycemic control (OR = 0.985; CI: 0.976–0.994; *p* = 0.002); moreover, the GA genotype of rs2167270 was protective against poor glycemic control compared to the GG genotype (OR = 0.417; CI: 0.245–0.712; *p* = 0.001). *Conclusions*: Higher serum leptin and the GA genotype of the rs2167270 SNP of the *LEP* gene were associated with good glycemic control in T2DM patients on metformin therapy. Further studies with a larger sample size from multiple institutions are required to validate the findings.

## 1. Introduction

Type two diabetes mellitus (T2DM) is a group of chronic, heterogeneous diseases that cause dysregulation of the metabolism of carbohydrates, fats, and proteins [1,2,3,4,5]. A consistent increase in blood glucose (hyperglycemia) is the characteristic feature of T2DM and forms the basis of its clinical diagnosis [6]. Evidence indicates that the pathophysiology of T2DM is complex and involves an intricate network of factors [6]. However, the major predisposing elements of disease development remain the presence of reduced insulin sensitivity in peripheral tissues, concomitant with a gradual loss of insulin secretion by pancreatic cells [6].

T2DM is a multi-systemic disorder with deleterious effects on most body tissues [7]. This is reflected by the plethora of complications associated with T2DM [7]. This disease increases the risk of atherosclerosis [8], renal failure [9], and blindness [10]. The complications above contribute to the high mortality rate of T2DM, which currently ranks as the 9th leading cause of death worldwide [11].

The lack of glycemic control in T2DM patients is one of the major predisposing factors for developing T2DM complications [12,13]. Accordingly, T2DM clinical practice guidelines have consistently emphasized the importance of maintaining blood glucose levels within a reference range to delay the progression of the disease and/or prevent its complications. For example, the American Diabetes Association (ADA) currently refers to an HbA1c percentage of 7% as the upper limit for good glycemic control in their practice guidelines [14]. In clinical and observational studies, compliance with the above guideline was shown to delay the progression of the disease and its complications [13,15], especially microvascular complications [16].

Despite being one of the cornerstones of T2DM management, many patients struggle to achieve glycemic control, and their HbA1c level remains above 7%. Several reasons could explain this conundrum in T2DM clinical care, including the genetic background of the patients [12] and their personality or behavioral traits [17,18,19]. Nonetheless, the reasons behind the lack of glycemic control remain a rich area for investigation [20].

Leptin is a hormone-like adipokine secreted by adipose tissue cells [21,22]. Inactivating mutations in the gene that codes for the leptin hormone (*LEP*) are linked with obesity in humans [23] and in rodent models [24]. Leptin causes an overall suppression of appetite and an increase in energy expenditure [25]. This explains why loss of leptin activity causes weight gain in animal models and in humans.

A growing body of evidence suggests that leptin plays a central role in regulating blood glucose levels [26,27]. For example, leptin was shown to enhance insulin sensitivity in its target tissues, causing an overall decrease in blood glucose [28]. In this context, it was shown that leptin could reduce glucose levels in hyperglycemic animal models that have low blood insulin levels [26], a metabolic state that resembles advanced stages of T2DM.

Leptin levels in the serum can also be affected by variations in the gene that codes for leptin (known as *LEP*). Indeed, several single nucleotide polymorphisms (SNP) in the *LEP* gene were associated with variations in leptin serum levels [29,30,31]. Given that variations in the leptin hormone itself are associated with variations in blood glucose levels, it was not surprising to observe that SNPs in the *LEP* gene were associated with differences in glucose levels and/or insulin resistance [32].

Based on the above observations, we tested whether variations in serum leptin levels could contribute to the lack of glycemic control in T2DM patients. Furthermore, we tested several SNPs in the *LEP* gene (rs7799039, rs2167270, and rs791620) for their association with poor glycemic control.

## 2. Materials and Methods

### 2.1. Study Design and Patient Recruitment

A hospital-based case-control study was conducted at King Abdullah University Hospital (KAUH), which is a tertiary teaching hospital that serves the northern governorate of Jordan. The hospital is affiliated with Jordan University of Science and Technology (JUST). The source population was all of the T2DM-confirmed cases attending KAUH. Study participants were those who presented at the diabetes clinic during the data collection period and who fulfilled the eligibility criteria described below. Cases were T2DM patients with poor glycemic control, while controls were T2DM patients with good glycemic control. The study involved 340 patients with a confirmed diagnosis of T2DM and a 1:1 case-to-control ratio. All patients recruited to the study were of Jordanian descent and were receiving treatment for T2DM at the time of their enrollment. The diagnosis of T2DM was according to the ADA clinical practice guidelines [14].

### 2.2. Eligibility Criteria

This investigation included patients receiving metformin alone without any other drug combination. Patients on insulin, sulfonylureas, or thiazolidinediones were excluded from the study. If the medical record indicated the presence of retinopathy, nephropathy, neuropathy, or atherosclerosis, patients were also excluded from the study. Other exclusion criteria included non-compliant patients. Compliance to treatment was judged by reviewing the refill history of the patients from the time of starting treatment at KAUH. Patients who missed four or more refills were excluded from the study.

### 2.3. Glycemic Control Definition

According to the guidelines of the American Diabetes Association (ADA) [14], T2DM patients with HbA1c levels of less than 7% were considered to have good glycemic control, whereas patients with HbA1c levels of 7 or more were considered to have poor glycemic control [33].

### 2.4. Ethical Approval

All patients enrolled in the study were required to sign a written informed consent. A summary of the study design and its objectives were verbally communicated to the patients by a clinical research coordinator prior to patient enrollment. This information was also listed on the consent signature page. The study was presented to the Institutional Review Board (IRB) of KAUH. The study was approved by the above IRB on the 27th of December 2018 with the following reference number: 7/119/2018.

### 2.5. Demographic, Anthropometric, and Clinical Data

T2DM patients who met the eligibility criteria above were requested to visit the diabetes clinic at KAUH. A clinical research coordinator interviewed the patient at the clinic and obtained informed consent. The age and sex of the patient were recorded. The weight (in kilograms (kg)), the height (in meters (m)), and the waist circumference (WC) in centimeters (cm) were measured and recorded as well. The measurement of WC was performed as described by Alfaqih et al. [34]. Height and weight measurements were used to calculate the body mass index (BMI) of the patient. The BMI value was calculated by dividing the weight in kilograms by the height in meters squared. The duration of treatment was defined as the length of time the patient has been receiving treatment for his/her condition since the time of their diagnosis. All the information above was entered into an Excel spreadsheet by a clinical research coordinator.

### 2.6. Blood Sample Collection

T2DM patients enrolled in the study were requested to visit the clinic following an overnight fast of 12-h duration. At 9:00 a.m. in the morning, two blood samples were collected. One sample of 5 mL of blood was collected into an EDTA tube (AFCO, Amman, Jordan). An aliquot of 1 mL of the blood from the above sample was stored at 4 °C for later use in DNA extraction. The remainder of the sample was sent to the biochemistry lab at KAUH to measure HbA1c levels. The biochemistry lab at KAUH utilizes an automated analyzer system (Roche Diagnostics, Mannheim, Germany) to measure HbA1c. The second sample (5 mL) was collected into a plain tube with a gel clot activator (AFCO, Amman, Jordan). This sample was used to obtain serum following centrifugation for 5 min at 4500 rpm. The resulting supernatant from the above centrifugation was equally distributed into four Eppendorf tubes that were frozen in liquid nitrogen and stored at −80 °C.

### 2.7. Biochemical Measurements

Glucose, cholesterol, and triglycerides were measured in the biochemistry lab of KAUH using serum samples stored at −80 °C. These measurements were made using an automated analyzer system (Roche Diagnostics, Mannheim, Germany). Leptin was measured using an enzyme-linked immunosorbent assay (ELISA) as described in Saadeh et al. [35]. Leptin ELISA kit was purchased from R&D Systems, Inc. (cat no. DY398; Minneapolis, MN, USA). Homeostasis model assessment-insulin resistance indices (HOMA-IR) were calculated using the following formula: (fasting insulin (μIU/L) × fasting glucose (mg/dL))/405.

### 2.8. DNA Extraction and Genotyping

Whole blood samples collected in EDTA tubes were used for the extraction of genomic DNA. Thermo Scientific Genomic DNA Purification Kits (cat no. K0512; Thermo Fisher Scientific, Inc.) were used in the procedure according to the instructions of the manufacturer. Following DNA purification, the final DNA yield was calculated based on DNA concentration measurements evaluated using an ND-2000 Nanodrop (Thermo Scientific, Waltham, MA, USA).

The polymerase chain reaction-restriction fragment length polymorphism (PCR-RFLP) technique was used for calling the genotype of the following SNPs in the *LEP* gene: (rs7799039, rs2167270, and rs791620). This technique uses PCR to amplify a genomic DNA fragment that contains the SNP, followed by restriction enzyme digestion of the resulting fragment. PCR reactions were 25 μLs in volume and contained 10 ng of genomic DNA, 0.4 μM of each of the forward and reverse primers (Integrated DNA Technologies, Coralville, IA, USA), and 1X of Taq Master Mix (New England Biolabs, Ipswich, MA, USA). The sequences of the forward and reverse primers used for genotyping each of the SNPs in the *LEP* gene can be found in Table 1.

**Figure 1:**
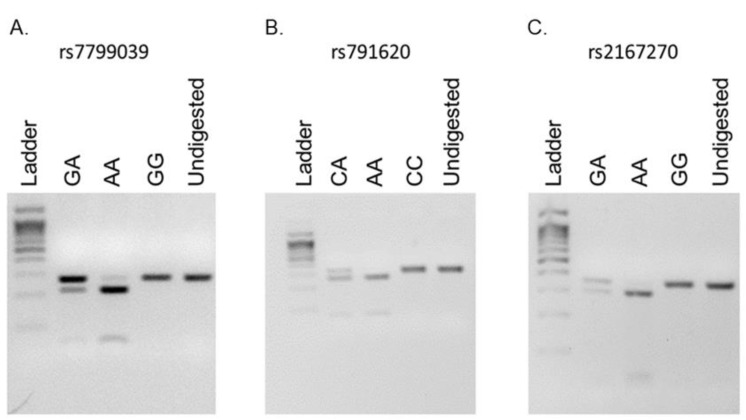
Genotyping strategy of *LEP* SNPs. A 3% agarose gel image of the different genotype classes of the (**A**) rs7799039, (**B**) rs791620, and (**C**) rs2167270 SNPs of the *LEP* gene observed in study subjects following PCR-RFLP.

Details of the PCR-RFLP assay (the location of the SNPs on the *LEP* gene and the size of the PCR amplicon) are summarized in Table 1. The name of the restriction enzyme used to genotype each SNP and the sizes of the PCR fragments that correspond to each of the different genotype classes of each SNP are also listed in Table 1. The DNA fragments that resulted from digesting the PCR product with the appropriate restriction enzyme were separated using 3% agarose gel electrophoresis (weight per volume). The gel was cast in the presence of ethidium bromide at a final concentration of 1 μg/mL. DNA fragments in the gel were visualized under ultraviolet light (see Figure 1). The genotype and allele call rates of all investigated SNPs were 100%.

### 2.9. Statistical Analysis

Statistical analysis was performed using SPSS version 28 (IBM Corp., Armonk, NY, USA). The normality of the data was checked using the Kolmogorov-Smirnov normality test (with a *p*-value ≥ 0.05 indicating a continuous variable with normal distribution). Statistical differences in age (which followed a normal distribution) were tested using an unpaired Student’s *t*-test. The Mann-Whitney U test was used to test the presence of statistically significant differences in BMI, WC, HbA1c, treatment duration, HOMA-IR, the serum levels of glucose, total cholesterol, triglycerides, or leptin between T2DM groups of good or poor glycemic control. Pearson’s *χ*^2^ test was used to assess the difference in gender distribution and was also used to examine the association between genotype or allele categories of each SNP with glycemic control.

Haplotype association analysis was performed using the SHEsis online platform (analysis.bio-x.cn/myAnalysis.php)(accessed on 14 February 2023). SHEsis is a robust and freely available platform used to estimate the haplotype frequency of genetic markers [36,37]. The above software employs computational models to estimate haplotype frequencies in a certain population using genotype data from individual genetic markers, including SNPs. The computational model employed by the SHEsis software uses a modified expectation-maximization algorithm to estimate haplotype frequency. This algorithm is known as Partition-Ligation-Combination-Subdivision EM [37]. The list of haplotypes and their frequencies were similar to the results obtained using the SNPstats platform (https://www.snpstats.net/start.htm)(accessed on 14 February 2023) [38], which employs the classical EM algorithm in its analysis.

Multivariate logistic regression models were performed to assess the independent association of several factors with glycemic control. The final model was selected using the forward LR (likelihood ratio) method, in which the most significant variables are added in a stepwise fashion to reach a sensitivity of 73.4% of the model. Odds ratios (ORs) and their 95% CI were estimated for each factor. A *p*-value < 0.05 (two-sided) was considered to be statistically significant for all analyses.

## 3. Results

### 3.1. Patient Characteristics and Biochemical Profile

This report included 170 T2DM patients classified according to their HbA1c levels into patients of good glycemic control or 170 patients classified into patients of poor glycemic control. The general characteristics of the patients in both groups are described in Table 2. Females comprised the largest percentage of both groups (62% and 54% of good or poor glycemic control, respectively). The mean age of the patients in both groups was around 58 years. No statistically significant differences were found for age, gender, BMI, or WC. Biochemical analysis showed that T2DM patients with poor glycemic control had significantly higher levels of serum glucose, HbA1c, and HOMA-IR and a longer treatment duration (*p* < 0.001). Total serum cholesterol or serum triglycerides, however, were not significantly different between the two groups (*p* > 0.05). Patients with poor glycemic control had significantly lower levels of serum leptin (*p* < 0.05). Serum levels of leptin remained significantly lower in patients with poor glycemic control following adjustment with BMI (*p* < 0.05).

### 3.2. Leptin and the GA Genotype of rs2167270 Reduce the Risk of Poor Glycemic Control

Patients in both groups were genotyped for three different SNPs in the *LEP* gene (rs7799039, rs2167270, and rs791620). The genotype and allele frequencies of each of the above SNPs are shown in Table 3 and Table 4, respectively. The analysis demonstrated significant differences in the frequencies of the rs2167270 genotypes between patients with good vs. poor glycemic control (*p* < 0.05). Specifically, patients with poor glycemic control had a higher frequency of the GG genotype (56.5% vs. 40.6%) but a lower frequency of the heterozygous GA genotype of rs2167270 (30.0% vs. 48.8%) (Table 3). No significant differences in the allele frequencies of the above SNPs were observed between the two groups (Table 4). The frequency of the A allele of rs2167270 was lower in patients with poor glycemic control (28.5% vs. 35.0%). The above difference did not reach statistical significance (*p* = 0.07) (Table 4).

In order to identify a chromosomal block in the *LEP* gene associated with glycemic control, we tested the association of different haplotypes resulting from genotyping all three SNPs of the *LEP* gene. The results of the above analysis are shown in Table 5. Herein, the GCA haplotype of rs7799039, rs2167270, and rs791620 had a lower frequency in patients with poor glycemic control compared to patients with good glycemic control. Accordingly, the GCA haplotype significantly lowered the risk of poor glycemic control (OR = 0.69, CI = 0.50–0.96, *p*-value = 0.03).

A logistic regression analysis was performed to investigate the effect of serum leptin, insulin resistance, treatment duration, and the genotypes of rs2167270 of the *LEP* gene on glycemic control status. The results showed that serum leptin remained significantly associated with glycemic control and reduced its risk (OR = 0.99; CI: 0.98–0.99; *p* = 0.002) (Table 6). Moreover, this analysis showed that HOMA-IR, or treatment duration were all significantly associated with a higher risk of poor glycemic control (*p* < 0.05) (Table 6).

Interestingly, using the multivariate model above, it was also observed that rs2167270 modified the risk of poor glycemic control. Specifically, the heterozygous GA genotype reduced the risk of poor glycemic control by aprroxiamtely 60% compared to patients carrying the GG genotype (OR = 0.42; CI: 0.25–0.71; *p* = 0.001) (Table 6).

## 4. Discussion

Good glycemic control in T2DM patients lowers the risk of developing micro- and macro-vascular complications and the risk of organ failure. Hence, the maintenance of good glycemic control was shown to reduce the mortality rate of T2DM [39]. Current practice guidelines recommend that adult T2DM patients have an HbA1c of <7% and a fasting blood glucose of <126 mg/dL [14].

For reasons that are not completely understood, a considerable fraction of T2DM patients never achieve the above goal [40]. In this report, we have shown that T2DM patients on metformin who lack glycemic control have lower levels of serum leptin following adjustment with age, BMI, treatment duration, and HOMA-IR. This finding highlights the tentative utility of several strategies (pharmacological or non-pharmacological) to achieve better glycemic control; these strategies will be discussed below.

Data on the correlation between serum leptin levels and glycemic control is scarce, especially in Jordan, a country in the Middle East and North Africa (MENA) region. In this report, we have shown that serum leptin was significantly associated with better glycemic control in T2DM, regardless of the patients’ BMI. In an interventional study, the use of insulin in T2DM patients who lack glycemic control and were originally on oral hypoglycemic drugs was associated with a reduction of HbA1c concomitant with an increase in serum leptin [41]. The above report provides indirect supporting evidence that the lack of glycemic control in T2DM patients is associated with lower levels of serum leptin, in agreement with our current findings.

The exact mechanism by which leptin could modify glycemic control in T2DM patients is unknown. The role of leptin in lowering serum glucose is well established [28]. The glucose-lowering effects of leptin could be indirectly attributed to its effect on decreasing food consumption and increasing energy use [42]. Another explanation could be that leptin is helping to achieve better glycemic control in T2DM patients by reducing the need for insulin secretion through an increase in insulin sensitivity [26,43].

A closer look at the progression of T2DM shows that there is an increasing number of patients who will eventually require insulin therapy later in the course of their disease, either alone or in combination with oral hypoglycemic agents [44]. However, insulin therapy in these individuals causes weight gain and could pose a higher risk of debilitating complications [45]. Based on the above, the introduction of treatment modalities alternative to (or in combination with) insulin therapy could be of utility to T2DM patients who stop responding to oral hyperglycemic agents. Buettner proposed “leptin therapy” as a superior alternative to insulin for the management of type 1 diabetes mellitus [46]. Noteworthy, data from pre-clinical animal models demonstrated the efficacy of leptin therapy in improving glycemic control in mice [28,47]. Hence, in theory, leptin therapy could be a viable approach to achieving better glycemic control in T2DM patients on metformin. This treatment modality could delay the introduction of insulin in these patients or allow the introduction of a lower dose of insulin. This, however, requires formal testing in well-designed clinical trials and is outside the scope of this report.

Leptin resistance is a metabolic state characterized by the failure of the body to increase energy consumption and suppress appetite despite an elevated level of serum leptin [48]. Leptin re-sensitization refers to dietary interventional strategies that increase the sensitivity of target tissues to leptin in obese individuals or in other individuals who have leptin resistance [49]. Given the above, it is conceivable that glycemic control in T2DM could be achieved via the re-sensitization of target tissues to existing leptin. The data presented in this report is preliminary and requires further validation. Nonetheless, our findings, if validated with a larger sample size and across multiple institutions, could trigger further research that attempts to employ the nutritional strategy described above alone or in combination with leptin therapy to achieve good glycemic control in T2DM patients. However, it should be emphasized again that the suggestion to implement such an intervention is still premature and requires formal testing.

The data shown in this report supports the idea that serum leptin is associated with glycemic control. Furthermore, we presented evidence that demonstrated a role for genetic variation in the gene that codes for leptin (*LEP*) in modifying glycemic control among the patients. Specifically, we found that the rs2167270 SNP in the *LEP* gene was associated with glycemic control in univariate and multivariate statistical models. This finding adds to the existing literature suggesting that glycemic control is the result of the complex interaction of a network of environmental and/or genetic factors [50].

In this report, it was observed that there was a significantly higher number of T2DM patients with good glycemic control who also carried the GA genotype of rs2167270 of the *LEP* gene. This result indicated that the GA genotype of rs2167270 reduced the risk of poor glycemic control. It was also found that patients with poor glycemic control have lower serum leptin levels. Interestingly, our group showed that Jordanian patients who have prediabetes, a state characterized by elevated levels of serum leptin, are more likely to carry the GA genotype of rs2167270 [21]. Taken together, it could be hypothesized that in Jordan, the GA genotype of rs2167270 is linked with metabolic conditions associated with elevated levels of serum leptin. This conclusion warrants further exploration using a larger population size in Jordan.

Dietary behavior could affect serum leptin levels or leptin resistance [51,52]. The research team did not collect any information relevant to differences in dietary behavior among the patients. This was one limitation of this current investigation. Another limitation was the relatively small sample size and the inclusion of patients from only one hospital.

## 5. Conclusions

This report represents one of the few studies that showed that a lack of glycemic control in T2DM patients on metformin therapy was associated with lower levels of serum leptin. Furthermore, it demonstrated that genetic variation in the gene that codes for leptin (*LEP*) modified glycemic control. These findings support the existing literature that glycemic control is the outcome of an interplay between the genetic background of the patients and/or the environment. In order to achieve glycemic control in T2DM patients, it is recommended to explore the benefits of utilizing therapeutic/nutritional strategies that reduce leptin resistance.

## Figures and Tables

**Table 1:** Information on the SNPs in the *LEP* gene and their genotyping strategy.

SNP ^1^ ID	Location and Base Change	Forward Primer Reverse Primer	PCR ^2^ Program (34 Cycles)	PCR Product Size (bp)	Restriction Enzyme, Incubation Temperature, and Time	RFLP ^3^ Product (bp)
rs7799039	Promoter (G/A)	GGGCTGGGAACTTTCTCTAAAG CCTGACTTCCTGCAACATCT	95 °C for 3 min, 35 cycles of 30 s at 95 °C, 30 s at 58.8 °C, and 1 min at 72 °C.	276	BtsIMutI, 55 °C, 1 h	GG: 276 GA: 276, 222, and 54 AA:222 and 54
rs2167270	5’ UTR (G/A)	CTGGAGGGACATCAAGGATTT AAGAAAGACCAGCAGAGAAGG	95 °C for 3 min, 35 cycles of 30 s at 95 °C, 30 s at 58.8 °C, and 1 min at 72 °C	348	HPYCH4III 37 °C, 1 h	GG: 348 GA: 348, 292 and, 56 AA: 292 and 56
rs791620	Regulatory region (C/A)	CTGGAGGGACATCAAGGATTT AAGAAAGACCAGCAGAGAAGG	95 °C for 3 min, 35 cycles of 30 s at 95 °C, 30 s at 63.0 °C, and 1 min at 72 °C	348	AscI 37 °C, 1 h	CC: 348 CA: 348, 262 and, 86 AA: 262 and 86

All SNP information was obtained from the NCBI dbSNP database; ^1^ SNP: single nucleotide polymorphis; ^2^ PCR: polymerase chain reaction; ^3^ RFLP: restriction fragment length polymorphism.

**Table 2:** Baseline characteristics of study groups.

Variable	Glycemic Control		*p*-Value ^1^
	Good *n* = 170	Poor *n* = 170	
Age (years)	58.88 ± 9.842	58.76 ± 10.05	0.91
Gender(n) (%) Male Female	65 (38%) 105 (62%)	79 (46%) 91 (54%)	0.12
BMI ^2^ (kg/m^2^)	30.50 (6.60)	30.60 (6.50)	0.57
WC ^3^ (cm)	106.00 (14.00)	106.00 (15.00)	0.28
Treatment duration (years)	3.00 (6.00)	8.00 (6.5)	<0.001
Cholesterol (mg/dL)	195.70 (79.60)	189.50 (93.80)	0.88
Triglycerides (mg/dL)	140.80 (104.00)	137.90 (119.20)	0.44
HbA1c ^4^	6.15 (0.82)	8.59 (2.17)	<0.001
Glucose (mg/dL)	147.40 (65.20)	231.60 (100.90)	<0.001
Insulin(pmol/mL)	23.10 (14.74)	29.66 (26.62)	<0.001
HOMA-IR ^5^	1.64 (0.77)	2.90 (2.44)	<0.001
Leptin (ng/mL)	34.24 (36.17)	26.41 (27.75)	0.008
Leptin/BMI	1.09 (1.08)	0.87 (0.85)	0.007

^1^ The *p*-values were calculated using the Mann-Whitney U test for non-normally distributed data and Student’s *t*-test for normally distributed data (age), except for gender distribution, which was calculated using Pearson’s chi-square; ^2^ BMI: body mass index; ^3^ WC: waist circumference; ^4^ HbA1c: glycated hemoglobin; ^5^ HOMA-IR: homeostatic model assessment insulin resistance. Data are presented as median (interquartile range) for non-normally distributed data, mean ± standard deviation for normally distributed continuous variables (age), and as n (%) for categorical variables (gender).

**Table 3:** Genotype frequencies of *LEP* SNPs in T2DM patients with good or poor glycemic control.

SNP ID	Genotype	Glycemic Control		*p*-Value ^1^
		Good (n = 170)	Poor (n = 170)	
rs7799039	GG GA AA	60 (35.3%) 86 (50.6%) 24 (14.1%)	58 (34.1%) 76 (44.7%) 36 (21.2%)	0.22
rs2167270	GG GA AA	69 (40.6%) 83 (48.8%) 18 (10.6%)	96 (56.5%) 51 (30.0%) 23 (13.5%)	0.002
rs791620	CC CA AA	160(96.5%) 10(6.0%) 0 (0.0%)	164(96.5%) 5(2.9%) 1 (0.6%)	0.26

^1^ *p*-values were calculated using Pearson’s chi-squared test.

**Table 4:** Allele frequencies of *LEP* SNPs in T2DM patients with good or poor glycemic control.

SNP ID	Allele	Glycemic Control		*p*-Value ^1^
		Good n (%)	Poor n (%)	
rs7799039	G A	206 (60.6%) 134 (39.4%)	192 (56.5%) 148 (43.5%)	0.28
rs2167270	G A	221 (65.0%) 119 (35.0%)	243 (71.5%) 97 (28.5%)	0.07
rs791620	C A	330 (97.0%) 10 (3.0%)	333 (98.0%) 7 (2.0%)	0.46

^1^ *p*-values were calculated using Pearson’s chi-squared test.

**Table 5:** Frequency of LEP haplotypes in T2DM patients with good or poor glycemic control.

	rs7799039	rs791620	rs2167270	Glycemic Control		OR ^1^ (95% CI ^2^)	*p*-Value ^3^
				Good (Frequency)	Poor (Frequency)		
1	G	C	A	0.35	0.27	0.69 (0.50–0.96)	0.03
2	A	C	G	0.38	0.42	1.18 (0.86–1.60)	0.31
3	G	C	G	0.24	0.27	1.23 (0.87–1.75)	0.24

^1^ OR: odds ratio; ^2^ CI: confidence interval; ^3^ *p*-values were calculated using Pearson’s chi-squared test. Only haplotypes with a frequency > 0.03 in T2DM patients with good or poor glycemic control are listed.

**Table 6:** Logistic regression analysis of study subjects.

Variable	OR ^1^ (95% CI ^2^)	*p*-Value ^3^
HOMA_IR ^4^	1.56 (1.31–1.85)	<0.001
Treatment duration (years)	1.12 (1.07–1.17)	<0.001
Leptin	0.99 (0.98–0.99)	0.002
rs2167270 GG GA AA	Reference 0.42 (0.25–0.71) 0.89 (0.40–1.97)	0.001 0.78

^1^ OR: odds ratio; ^2^ CI: confidence interval; ^3^ *p*-values were calculated using logistic regression analysis. ^4^ HOMA-IR: homeostatic model assessment for insulin resistance.

## Data Availability

The data presented in this study are available on request from the corresponding author.
